# Superior Ophthalmic Vein Access for Embolization of an Indirect Carotid Cavernous Fistula

**DOI:** 10.7759/cureus.1639

**Published:** 2017-09-01

**Authors:** Ali S Haider, Prabhat Garg, Dean Leonard, Tijani Osumah, Umair Khan, Steven Vayalumkal, Lyndon K Lee, Phu Nguyen, Grant Gilliland, Kennith F Layton

**Affiliations:** 1 Texas A&M College of Medicine; 2 School of Medicine, Ross University; 3 School of Medicine, St. George's University; 4 Texas A&m College of Medicine, Baylor University Medical Center; 5 Department of Radiology, Baylor University Medical Center

**Keywords:** carotid cavernous fistula, cavernous sinus thrombosis, embolization, superior ophthalmic vein

## Abstract

Carotid cavernous fistulae (CCF) are defined as abnormal connections between the carotid circulation and cavernous sinus. CCFs can be categorized as being direct or indirect. Direct CCFs are usually associated with trauma, whereas indirect CCFs are associated with revascularization following cavernous sinus thrombosis. We present a case of a 53-year-old male who presented with tinnitus, proptosis, conjunctivitis, and blurry vision. The patient had a recent endovascular transvenous embolization that was only partially successful, with a residual carotid cavernous fistula draining to the left superior ophthalmic vein and multiple cortical veins. A physical examination of the patient showed elevated intraocular pressures bilaterally. The patient had a high-flow indirect carotid cavernous fistula with bilateral superior ophthalmic vein (SOV) and retrograde cortical vein drainage. The SOV was punctured with a micropuncture needle and was used to successfully gain access to the cavernous sinus. Multiple coils were placed in the posterior aspect of the sinus until there was complete occlusion of venous flow. Coils were packed up to the posterior aspect of the orbit near the junction of the cavernous sinus with the SOV, and the embolization was successful. Indirect CCFs have gradual onset and are usually low-flow. Low-flow CCFs might improve with medical management.Some CCFs may cause ocular manifestations and can be symptomatically managed with prism therapy or ocular patching for diplopia, lubrication for keratopathy, or topical agents for elevated intraocular pressures. However, patients presenting with persistent ocular morbidity may require surgical or endovascular intervention.

## Introduction

Carotid cavernous fistulae (CCFs) are abnormal communications between the internal or external carotid arteries and the cavernous sinus. CCFs can be classified in a number of ways based on etiology (traumatic or spontaneous), rate of flow (low vs high), and the angiographic structure (direct or indirect). Most CCFs are direct communications between the internal carotid artery (ICA) and the cavernous sinus and present with ocular symptoms due to venous hypertension [1-2]. Indirect fistulae, on the other hand, characterize a much smaller proportion of CCFs and only rarely present with consistent symptoms. While indirect CCFs can sometimes be medically managed, direct CCFs tend to require invasive intervention, usually embolization of the cavernous sinus or ligation of the ICA. Here we present the case of a male with an indirect CCF and prior subtotal coil embolization of the cavernous sinus, who required definitive treatment with a superior orbital vein (SOV) cut-down for additional embolization. We further discuss the subtypes of CCF and the implications for treatment.

## Case presentation

**Figure 1:**
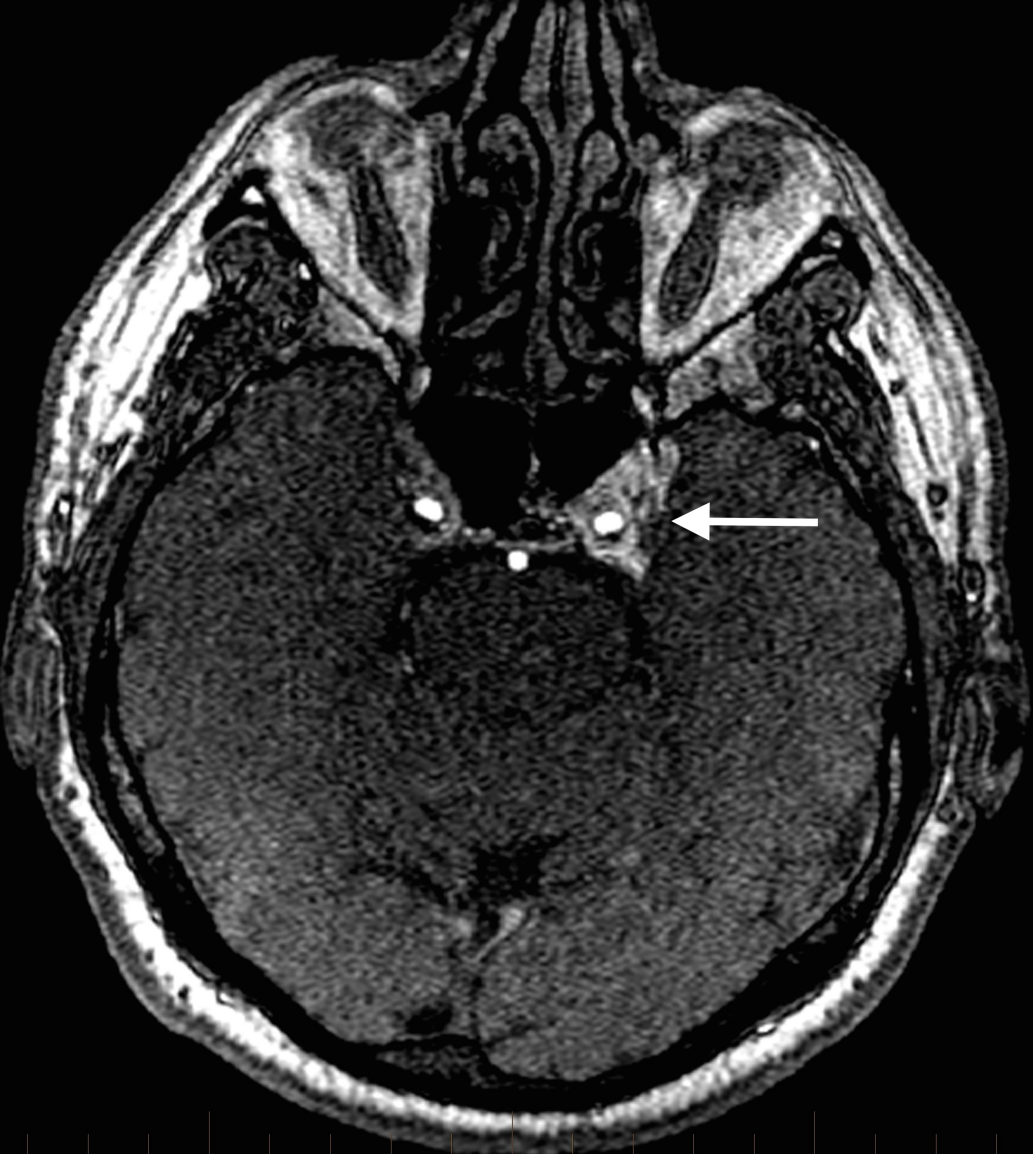
Axial source image from an intracranial magnetic resonance angiogram reveals abnormal arterial signal elevation in the left more than right cavernous sinuses consistent with a carotid cavernous fistula, as indicated by the arrow.

Our patient was a 53-year-old male with a high-flow indirect carotid cavernous fistula with bilateral SOV and retrograde cortical vein drainage. An initial intracranial magnetic resonance angiogram (MRA) revealed flow-related arterial signals in the left more than right cavernous sinuses (Figure 1).

**Figure 2:**
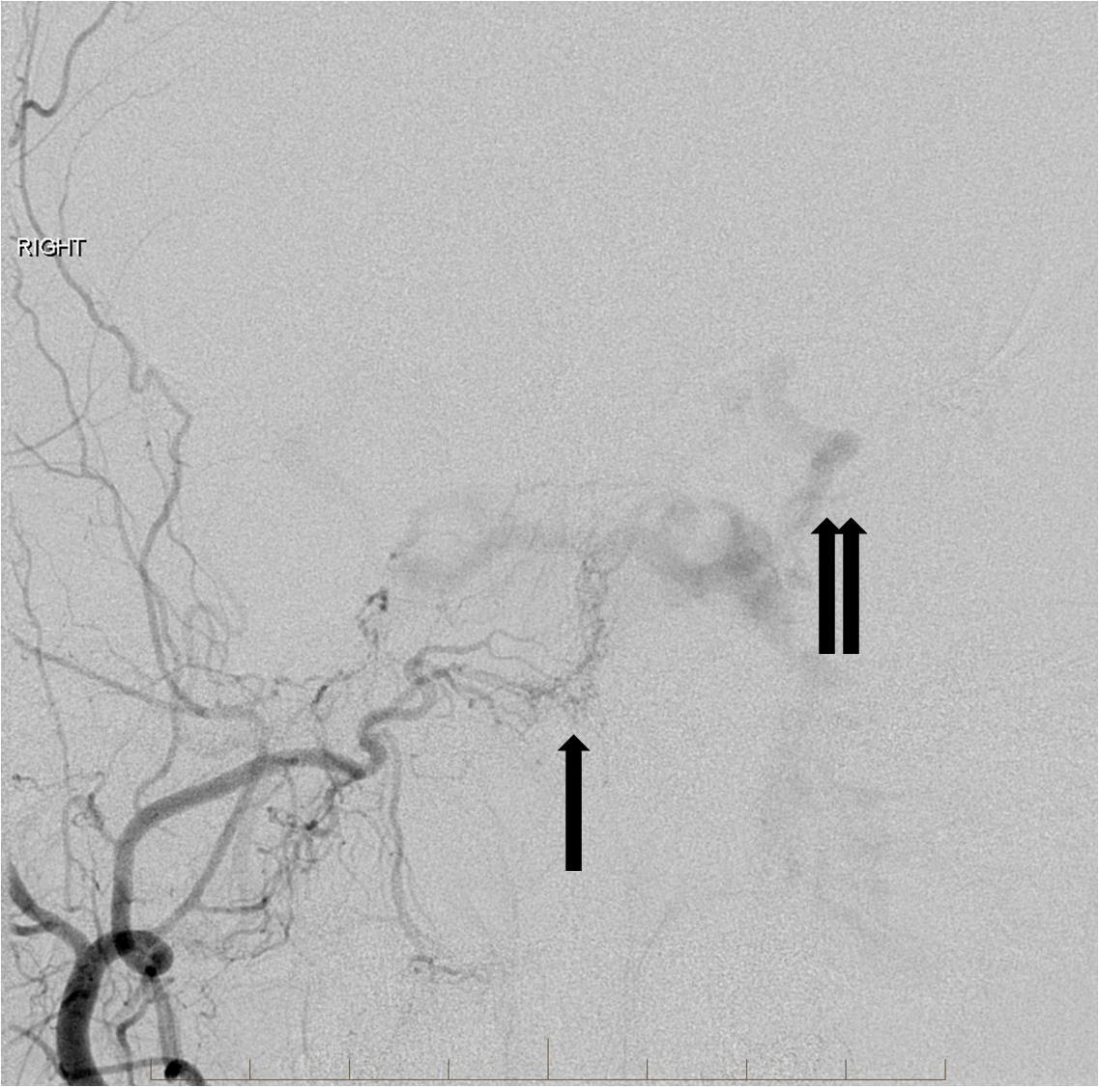
Right external carotid artery angiogram demonstrates multiple small feeding arteries to supply the indirect carotid cavernous fistula (single arrow). There is arterialization of the bilateral cavernous sinuses, circular sinus, and the left superior ophthalmic vein (double arrows). "Right" indicates the patient's right side.

**Figure 3:**
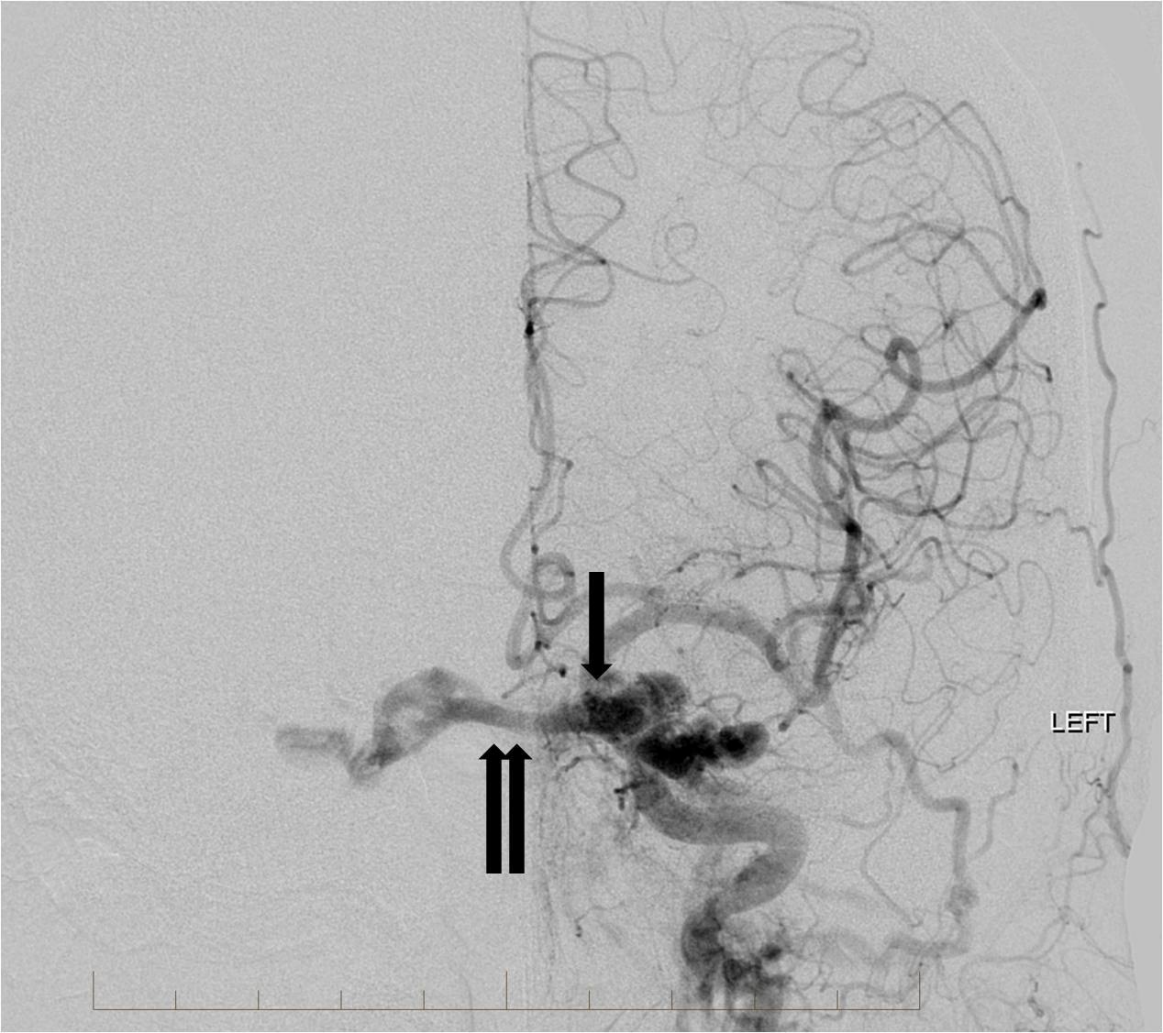
Frontal view from a left common carotid artery angiogram demonstrates multiple arterial feeders from the left internal carotid artery and external carotid artery to the high flow indirect carotid cavernous fistula (single arrow). There is arterialization of the bilateral cavernous sinuses, circular sinus, and left superior ophthalmic vein (double arrows). "Left" indicates the patient's left side.

**Figure 4:**
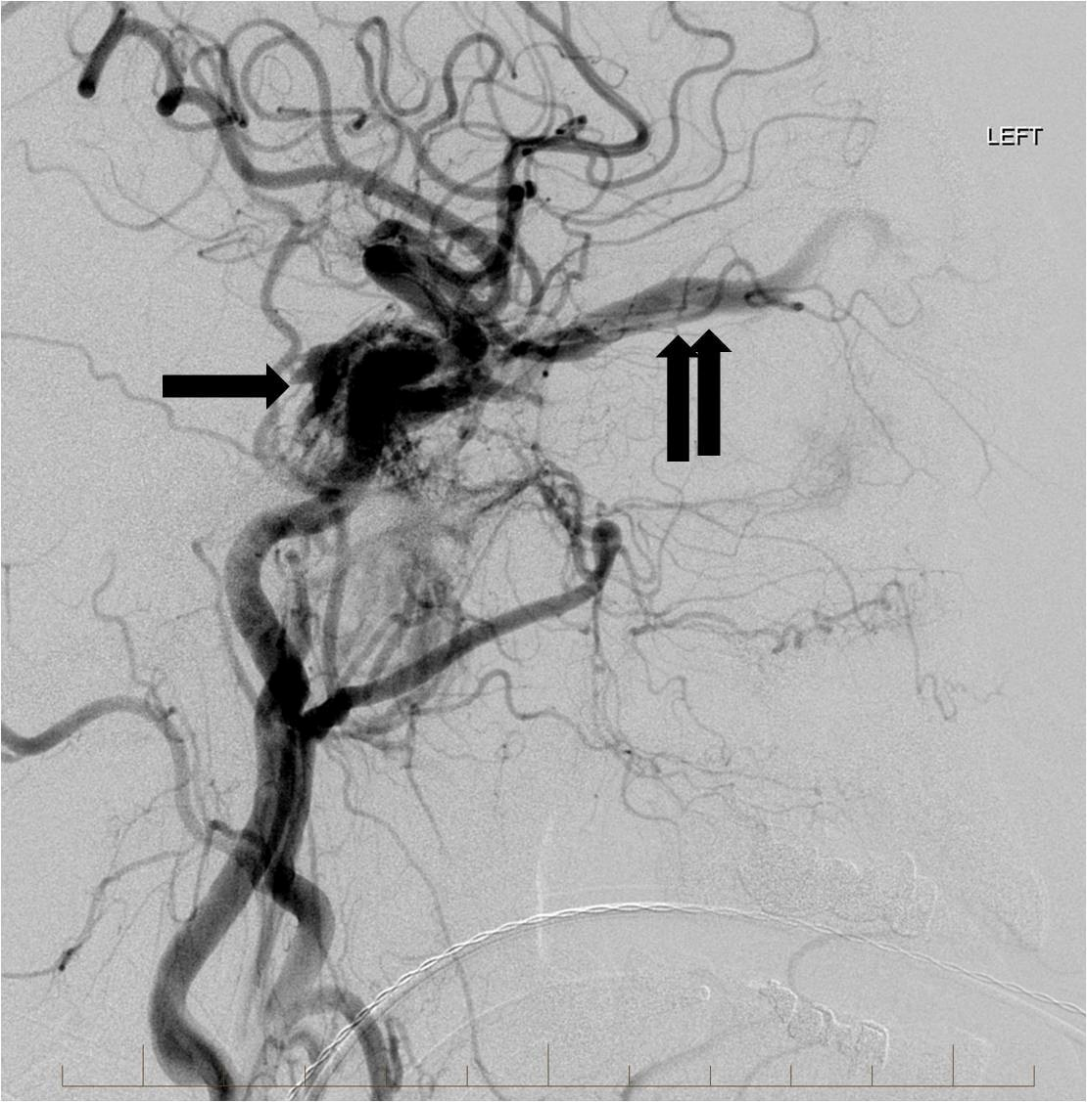
Lateral view from a left common carotid artery angiogram showing the high flow carotid cavernous fistula involving the cavernous sinus (single arrow) and left superior ophthalmic vein (double arrows). "Left" indicates the patient's left side.

His clinical presentation included tinnitus, proptosis, conjunctivitis, and blurry vision. A physical exam showed elevated intraocular pressures bilaterally. Arterial input was from innumerable small branches of both external and internal carotid arteries (Figures 2-4).

**Figure 5:**
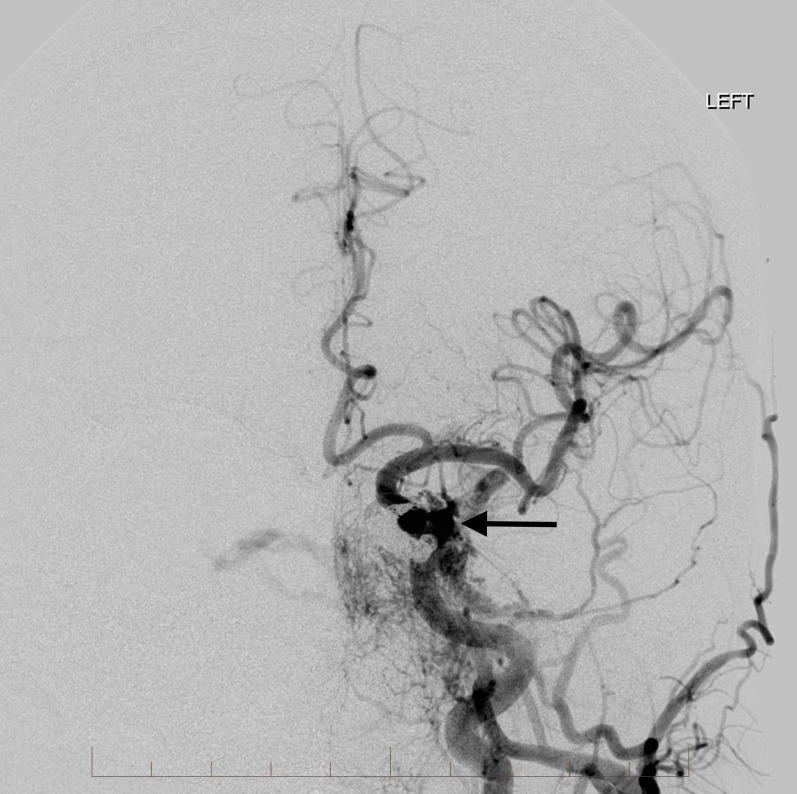
Left common carotid artery angiogram after the initial transvenous embolization reveals a dense coil pack in the medial aspect of the left cavernous sinus with reduced flow across the circular sinus. There is still a prominent arterialized venous pouch laterally (arrow) and arterialization of the left superior ophthalmic vein. "Left" indicates the patient's left side.

A recently attempted endovascular transvenous embolization was only partially successful with residual carotid cavernous fistula draining to the left SOV and multiple cortical veins (Figure 5).

**Figure 6:**
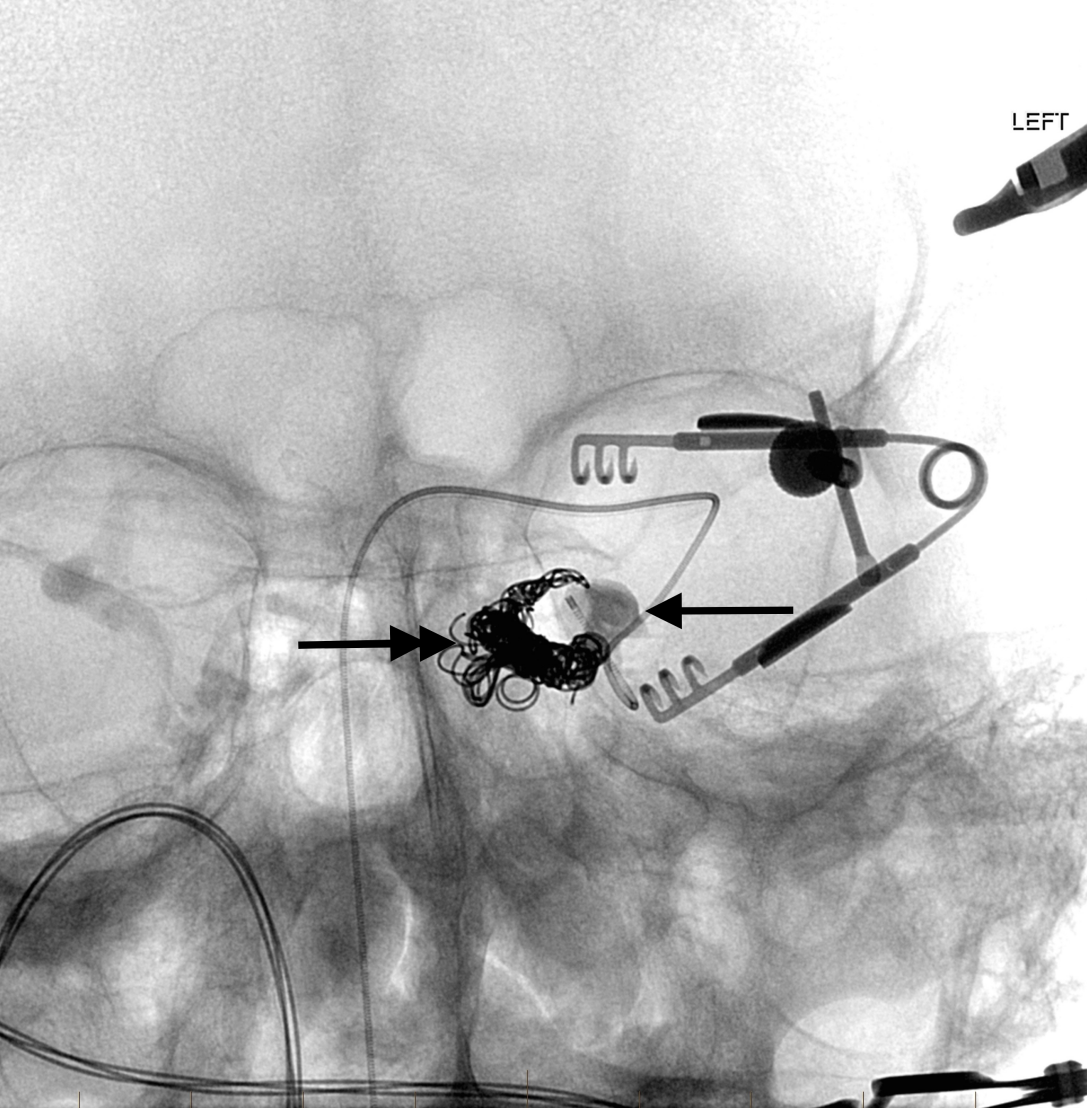
Frontal view native image after direct orbital cutdown for puncture of the left superior ophthalmic vein. Direct microcatheter venography opacifies the residual venous pouch in the lateral aspect of the cavernous sinus (arrow). Note the coils in the medial aspect of the sinus placed at the time of initial transvenous approach from the right facial vein (double arrow). "Left" indicates the patient's left side.

**Figure 7:**
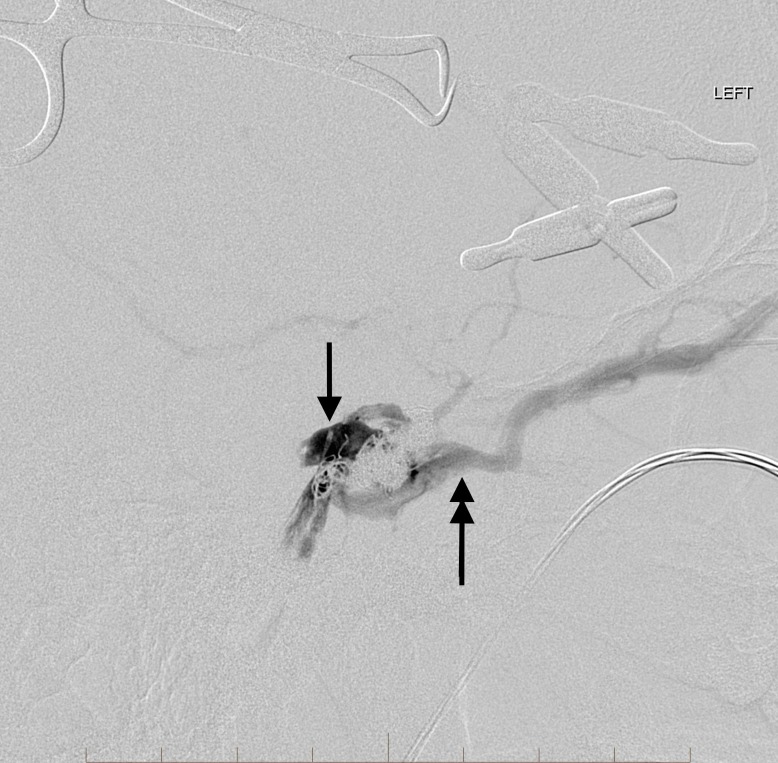
Lateral view selective microcatheter venography shows the microcatheter tip placed precisely in the residual arterialized venous pouch (arrow). Coils were placed from this position up to the junction with the superior ophthalmic vein (double arrow) as the microcatheter was slowly withdrawn. "Left" indicates the patient's left side.

On the initial embolization attempt, there was no communication between either inferior or superior petrosal or cavernous sinuses. The initial partial embolization required transvenous access through the right facial vein into the right SOV and across the circular sinus into the left cavernous sinus. As the left cavernous sinus had multiple septated compartments, only a portion of the left cavernous sinus could be accessed for embolization from the right transfacial venous approach. A subsequent encounter for transvenous embolization using a left orbital surgical cut-down approach to the SOV was planned to provide access to the cavernous sinus. The oculoplastic surgeon noted marked enlargement and arterialization of the SOV. The enlarged SOV was punctured with a micropuncture needle and was used to successfully gain access to the SOV with a 4 French micropuncture introducer. Through this, the neurointerventional radiologist advanced a 017 microcatheter over an 014 microwire into the isolated, residual lateral pouch in the left cavernous sinus. Super-selective left cavernous sinus venography was then performed (Figures 6-7).

**Figure 8:**
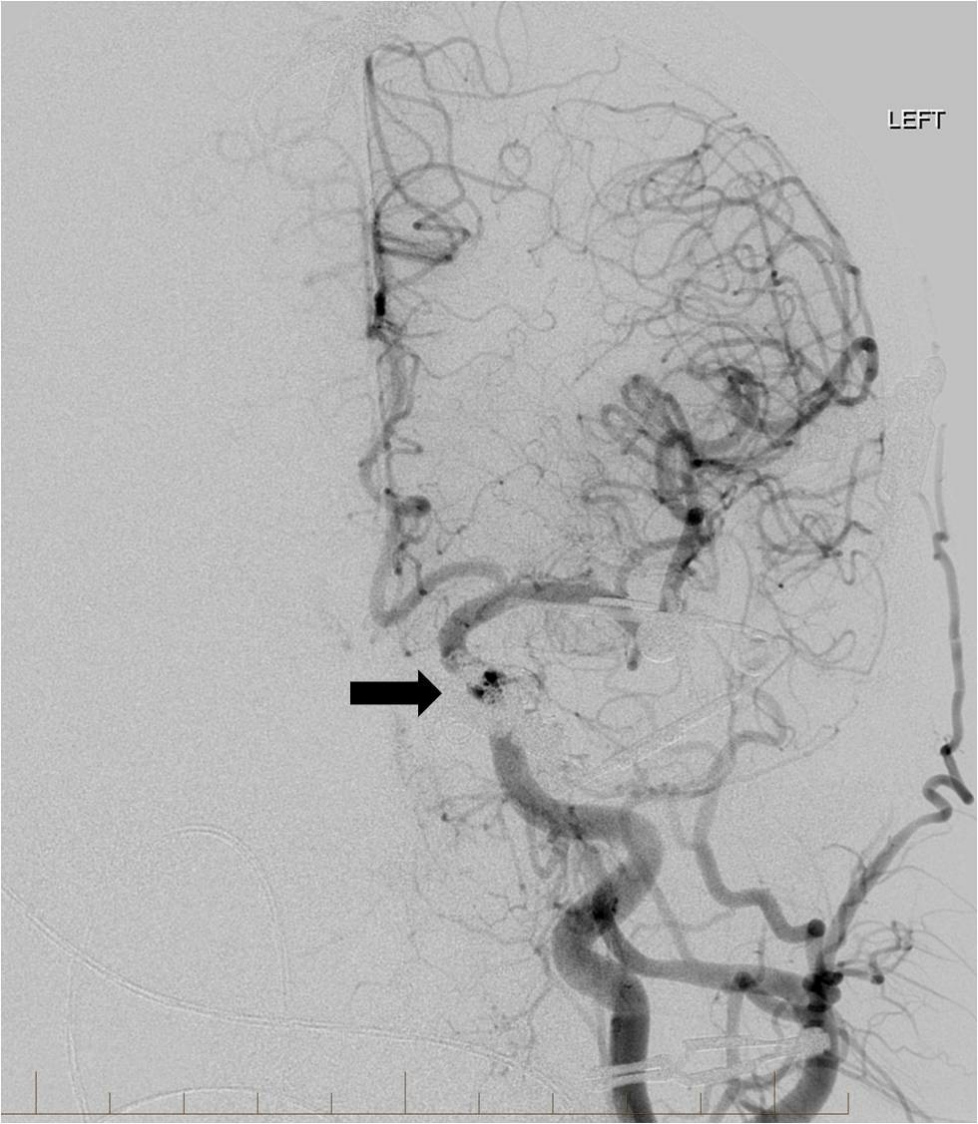
Final frontal view left common carotid artery angiogram shows elimination of the fistula after coil placement through the left superior ophthalmic vein cutdown approach. The arterialized cavernous sinuses, circular sinus, and superior ophthalmic vein are no longer visible (arrow). "Left" indicates the patient's left side.

**Figure 9:**
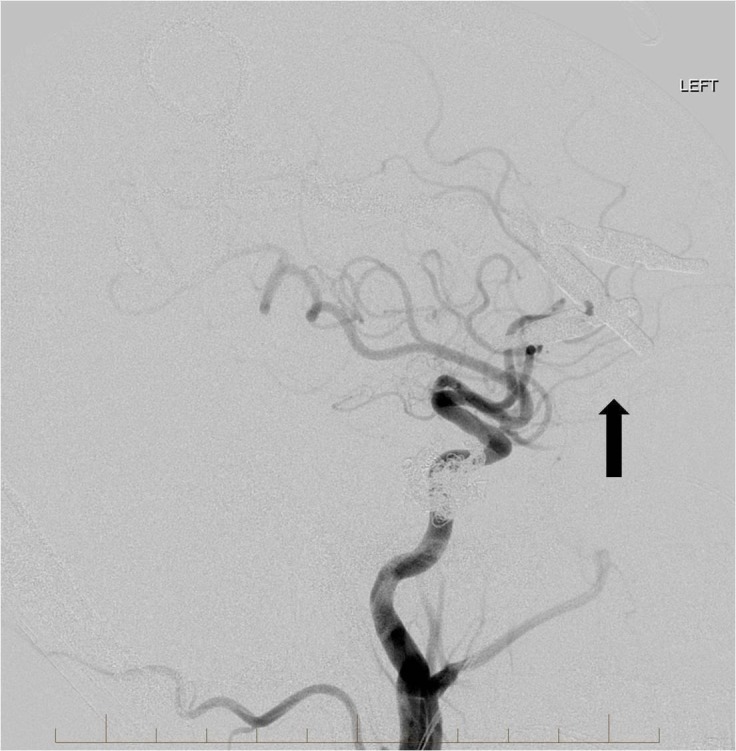
Lateral view left common carotid artery angiogram confirms that there is no longer any arteriovenous shunting or early opacification of the superior ophthalmic vein (arrow). "Left" indicates the patient's left side.

Multiple coils were placed in the posterior aspect of the sinus until there was complete occlusion of venous flow. Coils were packed up to the posterior aspect of the orbit near the junction of the cavernous sinus with the SOV, and the embolization was successful (Figures 8-9).

Bipolar cautery was used to maintain hemostasis and cauterize the remaining vascular malformation after careful blunt dissection to assure separation of the ocular muscles from the malformation. No residual bleeding was noted. The patient’s pupils remained within normal limits throughout the operation. Intraocular pressure was measured at 15 mmHg in the right eye and 17 mmHg in the left eye. The patient was taken to recovery without any complications. A follow-up four-vessel angiogram was performed five months after the operation, which showed no recurrent fistula.

## Discussion

Barrow, et al. classify CCFs using a four-point system: type A, B, C, and D [1]. Type A fistulae are direct communications between the ICA and the cavernous sinus. These usually consist of high flow rates. Type A fistulae are the most common and represent 70-90% of all CCFs. These generally affect young males and are usually traumatic, caused by penetrating trauma or motor vehicle collision [2]. Less frequently, these can also be caused by rupture of cavernous carotid aneurysms. Though most type A CCFs can be linked to a traumatic event, approximately 20% are spontaneous [3]. On the other hand, type B, C, and D are indirect fistulae that are mostly associated with dural arteriovenous fistulae (DAVFs). These fistuae are supplied by meningeal branches of either the ICA, the external carotid artery (ECA), or both, instead of a direct communication between the carotid system and the cavernous sinus. Type B and C fistulae are only supplied by the dural branches of the ICA and ECA, respectively. The most common indirect CCFs are type D fistulae, which are supplied by meningeal branches of both the ICA and ECA.

The classic clinical triad for a direct CCF with high flow is exophthalmos, bruit, and conjunctival chemosis. The fistula causes venous hypertension in ophthalmic veins leading to ocular signs and symptoms such as proptosis, chemosis, conjunctival injection, visual deficits, and cranial nerve paresis [4]. Patients with direct CCF can also develop bleeding from the mouth, nose, or ears. Additionally, 1-2% of patients can develop life-threatening epistaxis, while intracranial hemorrhage can manifest in 5% of patients due to retrograde arterialized cortical venous drainage. Unlike direct CCFs, indirect CCFs have gradual onset and usually are low-flow. The clinical presentation is generally milder and without the classical triad, though venous congestion can develop from sinus thrombosis. Abnormal arteriovenous shunting can lead to the development of recanalized dural veins and, because of the tortuous arterialization of the conjunctival veins, patients may develop chronic red eyes [5]. Due to the low-flow, some spontaneous indirect CCFs can improve with medical management [6]. Furthermore, some CCFs that cause ocular manifestations can be symptomatically managed with prism therapy or ocular patching for diplopia, lubrication for keratopathy, topical agents for elevated intraocular pressures, or even systemic steroids until the vascular lesions heal spontaneously [7]. However, patients presenting with persistent ocular morbidity may require surgical or endovascular intervention.

CCFs can be treated with several different options. Ligation of the cervical and intracranial ICA is one of them, which provides effective trapping of the fistula. However, such sacrifice of the ICA is performed sparingly due of the risk of cerebral infarction. Another approach is to embolize the cavernous sinus, preserving the ICA. This technique, however, puts the patient at risk of cranial nerve deficits [8]. Therefore, an endovascular approach is usually a better alternative to the traditional surgical approaches in managing CCF because of the removed risk of an open neurological surgery. Transarterial embolization with detachable balloons placed through the tear in the artery is the ideal treatment for a high-flow direct CCF [9]. However, due to some technical problems, these devices have been removed from the United States. Nevertheless, they continue to be used in many different parts of the world due to their simplicity and lower price. In the United States, transarterial embolization with coils or other embolic material is a common therapy for high-flow direct CCFs. Commonly used embolic agents are detachable platinum coils, n-butyl cyanoacrylate (n-BCA), and ethylene-vinyl alcohol copolymers (EVOH), such as the Onyx Liquid Embolic System (Medtronic, Dublin). Furthermore, self-expanding intracranial stents can be used to provide scaffolding to the injured ICA with subsequent coiling of the adjacent venous component. This prevents herniation of the coils from the cavernous sinus into the ICA [8]. In patients with extensive injury to the ICA, trapping the ICA may be the only option. However, before permanent occlusion, a temporary balloon occlusion study should be performed, if time permits, to better understand the risk and extent of cerebral infarction. Covered stent grafts can be an alternative option if the balloon test occlusion study is unsuccessful. These stents can effectively preserve the functional artery while eliminating the fistula. However, these stents have not gained FDA approval for intracranial use because of some disadvantages, namely, due to their stiff nature, navigating these stents through the tortuous petrocavernous ICA is difficult. Furthermore, they present an endoleak risk and coverage of vital perforators.

Transvenous endovascular management is the primary method of treatment for indirect CCFs. For low-flow indirect fistulae, transarterial approach can be cumbersome because of the small size, tortuous anatomy, and multiplicity of feeder arteries. In these patients, the transvenous approach is much simpler and carries a higher success rate. The goal is to catheterize the abnormal cavernous sinus very selectively and then occlude using embolic material. Though a transarterial approach for low-flow indirect CCFs is still an option for patients who fail the transvenous approach, access to the cavernous sinus is usually achieved through the inferior or superior petrosal sinus or through a facial vein communication. Our case presented several unique challenges. Our patient had an indirect fistula supplied with branches of both external and internal carotid arteries (type D). Additionally, this fistula was high-flow, which is uncommon for indirect fistulae. Due to the multicompartmental nature of the cavernous sinuses and limited venous access, only a partial occlusion was achieved from the initial transvenous approach. Since the left SOV was the major outflow from the fistula, we opted for a combined surgical and endovascular approach that allowed us to adequately reach the residual isolated arterialized pouch in the left cavernous sinus and eliminate both the cortical venous and left SOV outflow.

## Conclusions

It is important to remember that in some symptomatic indirect CCF cases, accessing the cavernous sinus can be extremely difficult due to venous outflow occlusion, stenosis, or marked tortuosity, as well as compartmentalization of the cavernous sinus. In these scenarios, a combined surgical and endovascular approach may be necessary to gain access to the cavernous sinus at the key site of fistulization. This access can be achieved either through a direct transorbital puncture or indirectly through the superior or inferior ophthalmic vein with the help of an experienced oculoplastic surgeon.
